# Going around the Kok cycle of the water oxidation reaction with femtosecond X-ray crystallography

**DOI:** 10.1107/S2052252523008928

**Published:** 2023-10-24

**Authors:** Asmit Bhowmick, Philipp S. Simon, Isabel Bogacz, Rana Hussein, Miao Zhang, Hiroki Makita, Mohamed Ibrahim, Ruchira Chatterjee, Margaret D. Doyle, Mun Hon Cheah, Petko Chernev, Franklin D. Fuller, Thomas Fransson, Roberto Alonso-Mori, Aaron S. Brewster, Nicholas K. Sauter, Uwe Bergmann, Holger Dobbek, Athina Zouni, Johannes Messinger, Jan Kern, Vittal K. Yachandra, Junko Yano

**Affiliations:** a Molecular Biophysics and Integrated Bioimaging Division, Lawrence Berkeley National Laboratory, Berkeley, CA 94720, USA; b Department of Biology, Humboldt-Universität zu Berlin, 10099 Berlin, Germany; c Molecular Biomimetics, Department of Chemistry- Ångström, Uppsala University, Uppsala SE 75120, Sweden; d Linac Coherent Light Source, SLAC National Accelerator Laboratory, Menlo Park, CA 94025, USA; e Department of Physics, AlbaNova University Center, Stockholm University, Stockholm SE-10691, Sweden; f Department of Physics, University of Wisconsin–Madison, Madison, WI 53706, USA; g Department of Chemistry, Umeå University, Umeå SE 90187, Sweden; Harima Institute, Japan

**Keywords:** photosystem II, oxygen evolving complex, manganese metalloenzymes, water-oxidation, water-splitting, X-ray free-electron lasers, X-ray spectroscopy

## Abstract

The water oxidation reaction in photosystem II (PS II) produces most of the molecular oxygen in the atmosphere, which sustains life on Earth, and in this process releases four electrons and four protons that drive the downstream process of CO_2_ fixation in the photosynthetic apparatus. The catalytic center of PS II is an oxygen-bridged Mn_4_Ca complex (Mn_4_CaO_5_) which is progressively oxidized upon the absorption of light by the chloro­phyll of the PS II reaction center, and the accumulation of four oxidative equivalents in the catalytic center results in the oxidation of two waters to di­oxy­gen in the last step. The recent emergence of X-ray free-electron lasers (XFELs) with intense femtosecond X-ray pulses has opened up opportunities to visualize this reaction in PS II as it proceeds through the catalytic cycle. In this review, we summarize our recent studies of the catalytic reaction in PS II by following the structural changes along the reaction pathway via room-temperature X-ray crystallography using XFELs. The evolution of the electron density changes at the Mn complex reveals notable structural changes, including the insertion of O_X_ from a new water molecule, which disappears on completion of the reaction, implicating it in the O—O bond formation reaction. We were also able to follow the structural dynamics of the protein coordinating with the catalytic complex and of channels within the protein that are important for substrate and product transport, revealing well orchestrated conformational changes in response to the electronic changes at the Mn_4_Ca cluster.

## Abstract 2

Photosystem II is the enzyme responsible for generating nearly all the oxygen in the atmosphere. Room-temperature time-resolved crystallography and spectroscopy measurements at X-ray free-electron lasers help to elucidate the sequence of events in the water oxidation reaction of photosystem II leading to the formation of di­oxy­gen from two molecules of water.

## 1. Introduction

**Figure 1:**
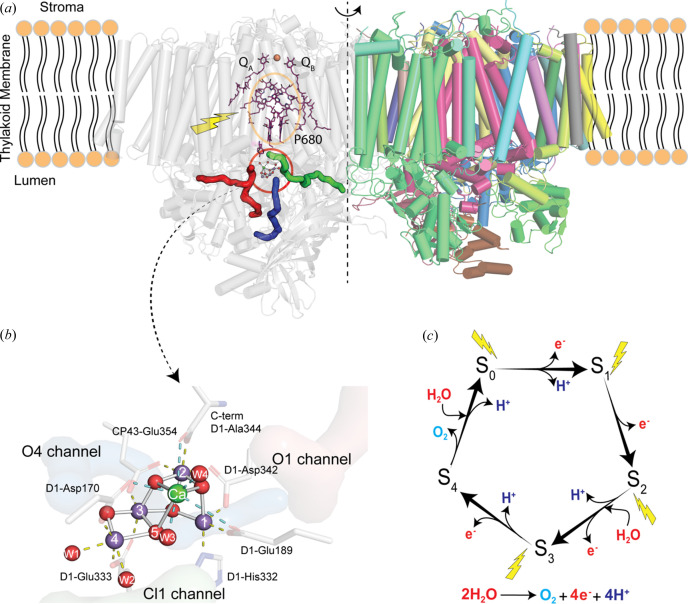
Overview of the water oxidation reaction in PS II. (*a*) Overall structure of PS II, embedded in the thylakoid membrane of cyano­bacteria, algae and higher plants. The PS II enzyme is a dimer with pseudo-*C*2 symmetry about the membrane axis as shown. Light absorption at the reaction center triggers charge separation that drives the water oxidation reaction at the oxygen evolving complex (OEC). The OEC (Mn_4_CaO_5_ cluster), where water oxidation takes place, is located on the lumenal side of the membrane. Three distinct water channels [depicted in red (O1 channel), green (Cl1 channel) and blue (O4 channel)] connect the cluster to the lumen and are thought to be involved in water and proton transport. (*b*) Detailed view of the OEC in the dark-stable S_1_ state with the coordinating waters and the water channels that extend towards the bulk from the catalytic center. Mn atoms are shown in purple, Ca atoms in green and O atoms in red. (*c*) The Kok cycle for the water oxidation reaction. Starting from the most reduced S_0_ state, each transition in the Kok cycle is initiated by the absorption of a photon of light by the reaction center in PS II, leading to the extraction of one electron from the OEC and advancement to the next S-state. Water oxidation and oxygen release take place after the formation of the transient S_4_ state and the cluster resets to S_0_.

In natural photosynthesis, the light-driven splitting of water into electrons, protons and molecular oxygen forms the first step of the solar-to-chemical energy conversion process (Shevela *et al.*, 2023 ▸; Cox *et al.*, 2013 ▸). The reaction takes place in photosystem II (PS II; Fig. 1 ▸), where the Mn_4_CaO_5_ cluster stores four oxidizing equivalents while cycling through its S_0_ to S_4_ intermediate states [Kok cycle; Fig. 1 ▸(*c*)] (Joliot *et al.*, 1969 ▸; Kok *et al.*, 1970 ▸; Yano & Yachandra, 2014 ▸). This cycle is driven by sequentially generated ultra-fast one-electron photochemical charge-separation reactions at the reaction center chloro­phylls. On charge separation, a pair of oppositely charged species is formed. The negative charge is transferred in the form of an electron to the acceptor side, consisting of electron acceptor quinones (*Q*_A_ and *Q*_B_) where a two-electron two-proton reduction takes place. On the other hand, the positive charge is transferred to the redox-active Tyrosine residue (Y_Z_) on the donor side of PS II. Neutralization of the positive charge on the Y_Z_ creates the main driving force for each transition in the Kok cycle. On reaching the S_4_ state after absorbing four photons, the oxidizing potential is used for the formation of the O—O bond and release of molecular oxygen. Thereafter, the system resets to its most reduced S_0_ state, the starting point for the next water oxidation cycle.

Elucidating the details of the water-splitting reaction in PS II is highly relevant for designing artificial photosynthetic systems from earth-abundant materials like Mn and the development of bio-inspired catalysts (Hunter *et al.*, 2016 ▸). To understand the mechanism of the water oxidation reaction in PS II, one of the strategies has been to follow the structural and chemical sequence of events in the enzyme by X-ray crystallography and spectroscopy using freeze-quenching of the stable intermediate states (Yano & Yachandra, 2014 ▸). By employing synchrotron methods, the ground state structure (S_1_ state) of PS II was obtained by several groups in the first decade at the turn of the millennium (Zouni *et al.*, 2001 ▸; Loll *et al.*, 2005 ▸; Kamiya & Shen, 2003 ▸; Ferreira *et al.*, 2004 ▸; Guskov *et al.*, 2009 ▸; Umena *et al.*, 2011 ▸). The high-resolution structure (1.90 Å) of the dark stable S_1_ state at cryogenic temperature by Umena *et al.* (2011 ▸) set the stage for further structural studies of higher S-states. However, PS II is very prone to radiation damage (due to the redox-active Mn_4_CaO_5_ cluster) from the X-ray beam (Yano *et al.*, 2005 ▸), and thus it is highly challenging to capture undamaged structures, especially of the higher S-states, using traditional crystallography methods at synchrotrons even under cryogenic conditions. We have been able to circumvent this problem via serial femtosecond X-ray crystallography (SFX) at X-ray free-electron lasers (XFELs) in the past decade (Alonso-Mori *et al.*, 2012 ▸; Kern *et al.*, 2012 ▸, 2013 ▸, 2014 ▸, 2018 ▸; Young *et al.*, 2016 ▸), leading to the determination of high-resolution structures for all stable S-states as well as time-resolved intermediates described in this paper. Other groups have also found similar success at XFELs for studying PS II with the first undamaged high-resolution S_1_ state structure at cryogenic temperature reported in 2015 (Suga *et al.*, 2015 ▸), and the S_3_ state structure reported in 2017 showing structural changes in the Mn cluster (Suga *et al.*, 2017 ▸). The X-ray pulses generated by an XFEL are extremely intense and with short pulse widths, containing as many photons (10^12^) in one pulse (<50 fs) as synchrotron beamlines provide in 1 s (Margaritondo & Ribic, 2011 ▸; Brändén & Neutze, 2021 ▸). The XFEL pulse is shorter (fs) than the time required for the diffusion of hydroxyl radicals (ps) caused by the X-rays in biological samples (Neutze *et al.*, 2000 ▸; Garman, 2010 ▸) and therefore the data can be collected before radiation-induced sample changes can develop, even at room temperature (see also Kern *et al.*, 2013 ▸; Fransson *et al.*, 2021 ▸). Thus, the train of XFEL pulses makes it possible to perform shot-by-shot X-ray data collection at room temperature, by synchron­izing XFEL pulses with the delivery of crystal suspension droplets, the visible laser triggers for photoexcitation and the detector readout (Fuller *et al.*, 2017 ▸). This capability has made it possible to follow the overall structural changes in PS II using X-ray crystallography and the chemical/electronic structural changes in the Mn_4_CaO_5_ cluster using X-ray spectroscopy while the catalytic reaction proceeds at room temperature (Kern *et al.*, 2018 ▸; Ibrahim *et al.*, 2020 ▸; Hussein *et al.*, 2021 ▸; Bhowmick *et al.*, 2023 ▸).

In this review, we summarize our recent studies of the structural changes at the oxygen evolving complex [(OEC) Mn_4_CaO_5_ cluster; Fig. 1 ▸(*b*)] and its environment during the Kok photosynthetic water oxidation cycle, by taking snapshots of the structures with X-ray crystallography under *operando* conditions (Bhowmick *et al.*, 2023 ▸; Hussein *et al.*, 2021 ▸; Ibrahim *et al.*, 2020 ▸; Kern *et al.*, 2018 ▸). Structures of the stable intermediate states of PS II as well as time-resolved states during the S_1_ → S_2_ transition have also been reported by other groups (Suga *et al.*, 2015 ▸, 2017 ▸, 2019 ▸; Li *et al.*, 2021 ▸). We first summarize our experimental methods that enable us to obtain the time-resolved crystallography data of PS II. We then discuss some of the key observations at the Mn_4_CaO_5_ cluster and its adjacent environment. Our XFEL studies emphasize the importance of a protein environment that can accommodate the charge density changes at the OEC and control the sequence of events during catalysis.

## 2. Crystallography data collection at XFELs

### 2.1. XFEL crystallography

The X-ray pulses generated at XFELs are ultra-short with pulse widths of 10–40 fs and are very intense (10^12^ photons). In comparison with synchrotron sources, XFEL pulses are about a billion times (10^9^) brighter. The high brilliance (tight focus and high intensity) allows us to study smaller crystals (<20 µm), opening the door to studying new types of materials that do not typically form large crystals (Spence, 2017 ▸; Barends *et al.*, 2022 ▸). The high intensity of the X-ray pulse however does mean that the crystal can be destroyed and thus the sample has to be replenished after each pulse. This has led to a completely different way of performing crystallography experiments. Instead of rotating the same crystal in the X-ray beam to collect diffraction data (traditional X-ray crystallography at synchrotrons), the sample has to be replaced continuously by some form of sample delivery. This way of collecting crystallographic data is often referred to as serial femtosecond crystallography (SFX) (Chapman *et al.*, 2011 ▸). Advances in sample delivery, detectors and data-processing technologies have led to major progress in SFX. Data from thousands of crystals can now be merged to yield a complete dataset at high resolution. Collecting data in a serial manner is also gaining ground at synchrotron sources around the world (Shilova *et al.*, 2020 ▸; Horrell *et al.*, 2021 ▸; Schulz *et al.*, 2022 ▸).

### 2.2. Time-resolved structural studies of PS II at XFELs

**Figure 2:**
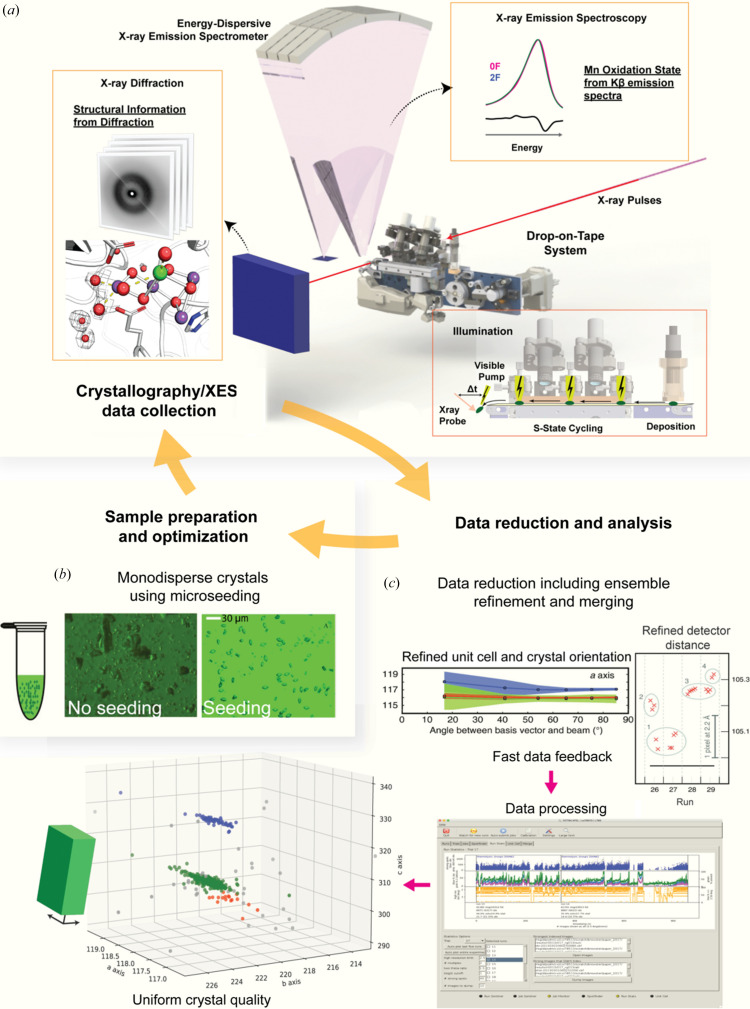
Components of the XFEL experiment. (*a*) Illustration of the setup in the experimental hutch with the drop-on-tape (DOT) sample-delivery method. The DOT method allows for precise illumination of the microcrystals including specific time-delay measurements with the free-space laser. The forward scattering leads to diffraction images, providing us with electron density maps and models. Measurement of fluorescence emission in the orthogonal direction provides us with spectroscopic information of the Mn cluster. (*b*) Microcrystals of PS II that have been optimized for XFEL experiments. Optimization steps include a seeding protocol for uniform size and a post-crystallization treatment protocol for isomorphous crystal batches. (*c*) Data-processing pipeline to obtain merged intensities from raw diffraction images. Precise merging of Bragg spots for PS II requires simultaneous refinement of the detector and crystal models to tighten their distributions. In the top box, the effect of the joint refinement is shown (blue: only crystal models from all shots are refined; green: crystal models and separate detector models for each shot refined; red: crystal models and single detector model across all shots refined). During live data collection, a fast-feedback mechanism via the *cctbx.xfel* GUI is used to provide a running analysis of the experiment, including spotfinding, indexing and merging statistics. In the lower box, a screenshot of the GUI is shown. In the top part, the blue trace shows the spotfinding statistics, in the middle part, the green trace shows the solvent hitrate, the blue trace shows the indexing rate and the pink trace shows the number of multiple lattices indexed. This feedback is used to further optimize sample conditions. Parts of (*b*) have been adapted from Ibrahim *et al.* (2015 ▸) and (*c*) from Brewster *et al.* (2018 ▸) and Brewster, Young *et al.* (2019 ▸).

The OEC is oxidized step-wise to higher S-states on absorption of a light flash (single photon) by the reaction center. Although the S_0_ state is the lowest oxidation state, the S_1_ state is the dark-stable state and the starting point for our time-resolved experiments. The enzyme progresses to the meta-stable S_2_ and S_3_ states after the first (1F, primarily S_2_ state) and second (2F, primarily S_3_ state) light flashes, respectively. Upon activation by the third flash (3F), one more oxidation of the cluster occurs (transient S_4_ state) and, subsequently, the cluster returns to the S_0_ state with the release of molecular oxygen. In order to study this reaction in a time-resolved manner at an XFEL, we have developed a new sample-delivery method (Fuller *et al.*, 2017 ▸), crystallization protocols (Ibrahim *et al.*, 2015 ▸; Hellmich *et al.*, 2014 ▸; Kern *et al.*, 2019 ▸) and data-processing techniques (Hattne *et al.*, 2014 ▸; Brewster *et al.*, 2018 ▸). These capabilities have allowed us to obtain electron density maps of PS II in an efficient manner with minimal sample wastage and extract maximum signal strength from the diffraction patterns. Importantly, the drop-on-demand delivery of PS II crystals in small droplets traveling on a conveyor belt allows precise illumination of the crystals with regards to timing and light intensity of the laser as well as minimizing double hits. This innovation has been crucial for obtaining high enrichment in the desired states of PS II to be studied, as verified by *in situ* XES and *ex situ* EPR studies on the crystals. An overview of our experimental setup is shown in Fig. 2 ▸. We summarize the various components of our PS II XFEL experiments in the following sections.

#### 2.2.1. Sample delivery: the drop-on-tape method

To take full advantage of the capability of XFELs for the mechanistic studies of PS II, delivering PS II crystals efficiently at the pulse repetition rate of XFELs with *in situ* photoexcitation is a critical requirement.

We developed a robust drop-on-tape (DOT) sample delivery setup for room-temperature X-ray crystallography/X-ray emission spectroscopy (XES), with photochemical triggers to advance the S-states *in situ* [Fig. 2 ▸(*a*)] (Fuller *et al.*, 2017 ▸). Droplets that contain protein crystal suspension are formed by an acoustic droplet ejector connected to the sample well that is re-filled via a Hamilton syringe driven by a syringe pump. Typically, the droplets are ∼150–250 µm in diameter and contain several dozen PS II single crystals (10–50 µm in size). With this setup, we routinely achieve a droplet hit rate of close to 100% and a crystal hit rate of ∼20–50%. With the focus of the XFEL beam of ∼2 µm, we typically obtain diffraction from no more than one single crystal from a droplet. Images with occasional multiple crystal hits can be deconvoluted and analyzed separately. The setup allows us to take snapshots of crystallography and XES data with a pump–probe time delay between ∼100 fs and up to several tens of milliseconds and we can advance PS II by exposing each single droplet to 1, 2, 3 or 4 light flashes before X-ray exposure. Importantly, the spacing between each of the initial light flash(es) as well as the final light flash is set to 200 ms, thus allowing full completion of the slower acceptor side reactions in PS II before exposure to the next light flash. In this paper, we use the convention of *n*F(Δ*t*) to denote time-point data where the sample is exposed to *n* (*n* = 1–4) light flashes and the XFEL beam hits the sample at time Δ*t* after the *n*th flash (Kern *et al.*, 2018 ▸). For intermediate S-states (S_*i*_, where *i* = 0–4), we use the standard nomenclature as shown in Fig. 1 ▸(*c*).

#### 2.2.2. Sample preparation and crystal uniformity

The PS II microcrystals (10–50 µm) for XFEL measurements were prepared from PS II dimers isolated from the thermophilic cyano­bacterium *Thermosynechococcus vestitus* (previously named, *Thermosyncechococcus elongatus*) using the detergent octa­ethyl­ene glycol monodo­decyl ether (C_12_E_8_) for protein extraction and purification (Hellmich *et al.*, 2014 ▸). PS II crystal suspensions were loaded into a syringe and dark-adapted for 1 h before data collection. Membrane inlet mass spectroscopy (MIMS) was used to determine the O_2_ evolution activity, turnover parameters and S-state populations of each sample batch prior to the experiment (Bhowmick *et al.*, 2023 ▸; Ibrahim *et al.*, 2020 ▸; Kern *et al.*, 2018 ▸). The PS II crystals showed no Mn(II) contamination based on EPR (*ex situ*) (Kern *et al.*, 2018 ▸) and XES (*in situ*) measurements and exhibited high activities (Fransson *et al.*, 2018 ▸). Early XFEL studies of PS II yielded crystal structures with a resolution in the range 6.5–4.5 Å (Kern *et al.*, 2012 ▸, 2014 ▸). To improve the diffraction quality of the microcrystals, we have developed a seeding protocol that leads to a more uniform shape and size of PS II crystals [see Fig. 2 ▸(*b*)] (Ibrahim *et al.*, 2015 ▸). This leads to a more stable sample ejection and higher-quality diffraction patterns. In PS II microcrystals, the diffraction quality also depends on the unit cell of the crystals. Thus, we optimized experimental conditions to maximize the population of PS II crystals that give us the highest-resolution images and also worked on obtaining highly isomorphous crystal batches, which is imperative for making comparisons between different states and for generating difference maps. A key step here is to precisely adjust the final detergent concentration in the crystals to a lower value. For this, we developed a post-crystallization treatment protocol (Hellmich *et al.*, 2014 ▸) which removes the detergent from the PS II crystals, leading to a more native packing of the crystal lattice, highly isomorphous crystal batches and consequently better diffraction (Kern *et al.*, 2019 ▸). In Fig. 2 ▸(*b*), we show the unit-cell dimensions obtained for individual PS II crystals. The graph shows that these predominantly cluster into three isoforms of the unit cell with the most optimal one in terms of diffraction quality being the crystal form colored in green. Owing to their non-isomorphism, crystals that do not belong to this cluster are excluded when calculating structure factors. With these improvements, we can now collect datasets consistently at about 2 Å resolution. Typically, each dataset requires about 4–5 ml of sample, yielding 20 000–30 000 diffraction images.

#### 2.2.3. Data processing

The data collected for the different illumination states were processed using the program *dials.stills_process*. Bragg spots were integrated to the edge of the detector. A kapton absorption correction for the shadow produced by the tape of our sample delivery system was applied to each integrated Bragg spot, taking into account the droplet size, tape thickness, tape angle and the position of the diffraction spots on the detector with respect to the crystal position (Fuller *et al.*, 2017 ▸). Prior to integration, we also performed an ensemble refinement of the crystal and detector parameters using the program *cctbx.xfel.stripe_experiment* which has been shown to narrow the unit-cell and detector distance distributions [see Fig. 2 ▸(*c*)] and improve the final isomorphous difference maps (Brewster *et al.*, 2018 ▸). Finally, the intensities were merged using the program *cctbx.xfel.merge* (Sauter, 2015 ▸; Brewster, Bhowmick *et al.*, 2019 ▸) which applies a per-image resolution cutoff and filtering of the lattices using a unit-cell threshold of 1% from the reference model. An important aspect of performing the experiment is fast feedback from the data-processing side to make swift experimental decisions about sample conditions (to optimize crystallization and post-crystallization treatment) and stability of the ejection system. For this purpose, a graphical user interface (GUI) has been developed which allows non-experts to process and track the experimental results (Brewster, Young *et al.*, 2019 ▸). This includes tracking the hit rate, indexing rate and unit-cell distribution. The software has also incorporated multi-processing capabilities which can leverage supercomputing resources to provide feedback in real time (Blaschke *et al.*, 2024 ▸). Once structure factors are obtained, refinement, model adjustments and map calculation are carried out using *phenix.refine* (Liebschner *et al.*, 2019 ▸) and *Coot* (Emsley & Cowtan, 2004 ▸) downloaded from their respective servers. Custom restraints for the OEC, developed from data available from spectroscopy measurements, were used during structural refinement [see Bhowmick *et al.* (2023 ▸) for details].

### 2.3. Characterization of the S-states by spectroscopy

The XFEL data collected for the various flash states, especially the 2F (S_3_) and the 3F (S_0_) data, are a mixture of different pure S-states. This is due to the intrinsic turnover inefficiency of the various S-state transitions (Han *et al.*, 2022 ▸) that lead to de-synchronization of the various centers in the crystal. While the 0F and 1F states are dominated by the S_1_ and S_2_ states (each populated to >90%, respectively, in these samples), the 2F (majority S_3_) and 3F (majority S_0_) states are more mixed with the S_3_ population in the 2F state being 75% and the S_0_ population in the 3F state being 60%. The pure S-state populations of these flash states can be disentangled by multiple methods including *ex situ* MIMS (Beckmann *et al.*, 2009 ▸) and EPR spectroscopy (Kern *et al.*, 2018 ▸) as well as *in situ* XES (Fransson *et al.*, 2018 ▸, 2021 ▸) carried out simultaneously with X-ray crystallography. More details about these measurements as applied to the XFEL experiment can be found in the work by Kern *et al.* (2018 ▸). In the crystallographic model, this mixture of population is accounted for by constructing a multi-component model for the S_2_ → S_3_ and the S_3_ → [S_4_] → S_0_ transitions. For the rest of the review, we only show results from the component that is undergoing the transition of interest. Henceforth, we also refer to the S_3_ → [S_4_] → S_0_ transition as the S_3_ → S_0_ transition.

## 3. Going around the Kok S-state clock

### 3.1. Structural changes at the OEC

**Figure 3:**
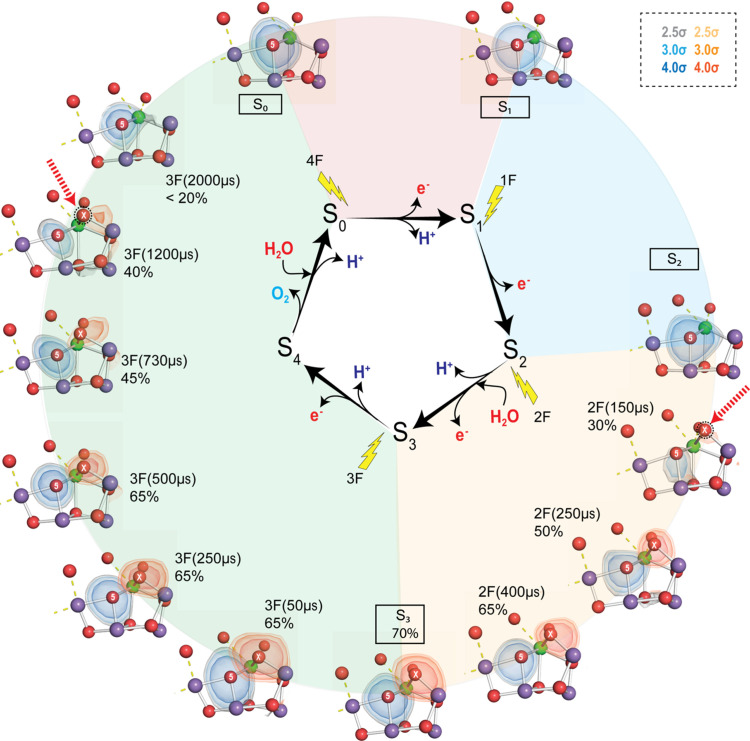
Changes in the electron density at the OEC during the Kok cycle. The omit map of the oxygen atom O_X_, which is inserted during the S_2_ → S_3_ transition as a bridging oxygen between Mn1 and Ca, is shown in orange. For reference, omit maps constructed by separately omitting the O5 atom are overlaid in blue at the same density levels. The density of O_X_ increases gradually in the S_2_ → S_3_ transition and starts to decrease after 500 µs in the S_3_ → S_0_ transition with the density below noise level by 2000 µs. The red arrows show the first and last time points where we observe significant O_X_ density. The populations (%) shown are modeled O_X_ occupancies [except at 3F (2000 µs)] in the primary component (see text). Note that because we show the primary component here, the O5 and OEC populations are also changing. Mn atoms are shown as purple, Ca atoms as green and O atoms as red spheres. Color legend for the various σ levels of the omit maps shown are provided in the top-right box.

Fig. 3 ▸ shows the changes at the OEC during the PS II reaction cycle. In order to best show the changes in the electron density of light atoms like oxygen in the vicinity of heavier scatterers like Mn, we use omit maps of the individual light atoms. Here we show the omit map of O5 (in blue) which is coordinated to Mn3/Mn4 and Ca [see Fig. 1 ▸(*b*) for the numbering of atoms in the Mn_4_CaO_5_ cluster]. We also show the omit map of the new oxygen ligand O_X_ [in orange, also called O6 in the literature (Suga *et al.*, 2017 ▸)] which appears during the S_2_ → S_3_ transition, bridging Mn1 and Ca, and disappears during the S_3_ → S_0_ transition. At the start of the reaction cycle (S_1_ state), the Mn cluster is in the (III, IV, IV, III) formal oxidation state (corresponding to Mn1, Mn2, Mn3, Mn4) in which the Mn1 atom is penta-coordinated with an open sixth coordination site. This is confirmed by the Mn1—O5 distance, which is 2.8 Å whereas the Mn4—O5 distance is 2.1 Å, thus ruling out a bond between Mn1 and O5. On transition to the S_2_ state (1F), the OEC geometry remains fundamentally unchanged. It is thought that the Mn4 atom is oxidized from +III to +IV because of the shortening of the Mn4—O_D1–E333_ distance as discussed by Ibrahim *et al.* (2020 ▸) and Krewald *et al.* (2015 ▸).

Major changes in the cluster can be seen upon application of the second flash (2F). About 150 µs after the second flash, we begin to observe the emergence of a new water molecule in the cluster, O_X_. This new ligand [O_X_, also called O6 by Suga *et al.* (2017 ▸)] is inserted into the open coordination site of Mn1 as a bridge between Ca and Mn1 (Fig. 3 ▸). This insertion also leads to an expansion of the cluster, most prominently seen in the Mn1—Mn4 distance which is a key surrogate measure for the presence of O_X_. Because of the higher scattering power of Mn, metal–metal distances are the most reliable indicators of major changes happening at the cluster. The distance increases from 4.8 Å in S_1_ to 5.2 Å in the 2F(150 µs) dataset before stabilizing at 5.05 Å in the S_3_ state. The omit map suggests that most of the O_X_ insertion is complete by 400 µs. The increase in density of O_X_ tracks well the oxidation state of the cluster, probed by Mn *K*β XES measurements on PS II solution samples (Kern *et al.*, 2018 ▸; Ibrahim *et al.*, 2020 ▸). This suggests that the oxidation of the cluster, and more specifically Mn1, and the water insertion into its sixth coordination site are highly correlated events. The time-constant for this reaction is estimated to be ∼350 µs from the XES studies. On completion of this transition, the S_3_ state is formed and the Mn cluster is formally in the (IV, IV, IV, IV) state (Cox *et al.*, 2014 ▸). It has been shown that the charge density on the Mn atoms is more delocalized as the cluster becomes more oxidized. All Mn sites at this stage are six-coordinate.

Upon the third flash (3F), the S_3_ → S_0_ transition is initiated. This reaction is known to be the slowest step in the Kok cycle with a time-constant τ ≃ 1.1–2 ms depending on the type of the samples, experimental conditions used to study the reaction and the kinetic process probed by the spectroscopy method (Rappaport *et al.*, 1994 ▸; Razeghifard & Pace, 1997 ▸; Renger, 2012 ▸; Noguchi, 2015 ▸; Zaharieva & Dau, 2019 ▸). This is the step where molecular oxygen is formed and the cluster is reset to the S_0_ state, which also involves the insertion of a water molecule and the removal of two protons. Given this context, we expect to see distinct changes in the OEC associated with these processes during this transition. From our XFEL crystallography data (Bhowmick *et al.*, 2023 ▸), we observe that the densities of O5 and O_X_ do not change substantially until 500 µs after the application of the third flash. The most prominent change is first seen at 500 µs when the density shape of O_X_ changes, showing an asymmetry towards O5. In the next time point (730 µs), the density is substantially reduced and is even more anisotropic. By 1200 µs, the O_X_ density is close to the noise level of the map (2.5–3σ) and is well below the noise level by 2000 µs. Thus, the structural data show a gradual reduction in the O_X_ population, starting from about 500 µs to about 1200–2000 µs after the third flash. For O5, we only see a prominent dip in the density around 1200 µs which is subsequently restored by 2000 µs. Interestingly, the O5 density during the 730–1200 µs interval appears quite asymmetric, probably encompassing other neighboring ligand atoms. The Mn1—Mn4 distance contracts at 2000 µs, returning to 4.97 Å, similar to the S_0_ state. This provides further evidence for the disappearance of O_X_ around this time point. The structural data for the S_3_ → S_0_ transition at the OEC is starting to provide us with clues on the order of events leading to the formation of molecular oxygen and subsequent resetting of the Mn cluster for the next reaction cycle. The current data, as discussed by Bhowmick *et al.* (2023 ▸), suggest that O_X_ is either involved in the O—O bond formation with O5 in the S_3_ → S_0_ step or it replaces O5 after the formation of molecular oxygen. In the second case, O5 could form an O—O bond with either W2 or W3. Future XFEL studies should shed more light on the exact mechanism of O—O bond formation. This includes collecting higher-resolution datasets and data at additional time points that would give us a more fine-grained picture of the events leading to O—O bond formation.

### 3.2. Structural changes in the amino acid coordination of the OEC

**Figure 4:**
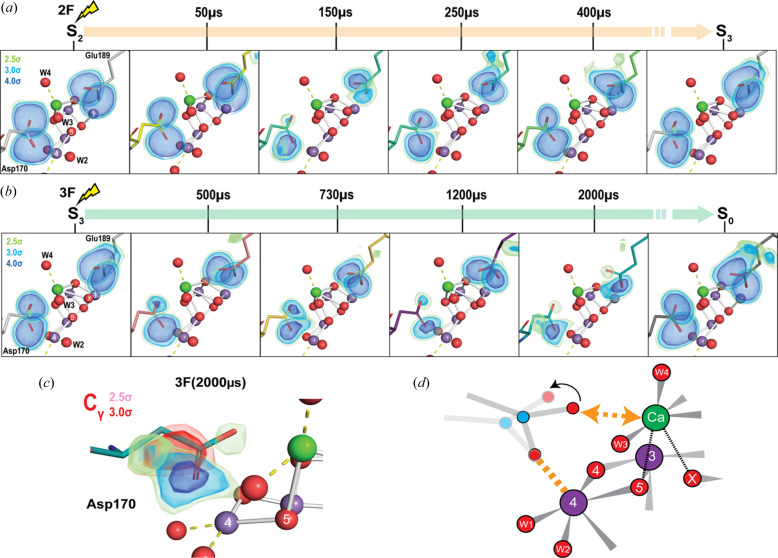
Time-resolved motions in the coordinating ligands of the OEC. The protein environment near the OEC appears to undergo conformational changes in response to the oxidation of the cluster after each light flash. Shown are the omit map densities of each of the carboxyl­ate oxygens in D1-Asp170 and D1-Glu189 in the (*a*) S_2_ → S_3_ and (*b*) S_3_ → S_0_ transitions, and (*c*) a more detailed omit map density of each of the carboxyl­ate oxygens and the C_γ_ atom of Asp170. For the omit maps of the carboxyl­ate oxygens, the same color legend to depict the σ levels used in (*a*) and (*b*) have been used. The color legend for the C_γ_ atom omit map is provided in the figure. (*d*) Possible model for the movement of Asp170, suggesting it could be detached from the Ca for certain time points in both transitions in synchronization with the water insertion event. Mn atoms are shown as purple, Ca atoms as green and O atoms as red spheres.

The Mn_4_CaO_5_ cluster is located in a highly negatively charged enzyme pocket with multiple amino acids from the D1 subunit and one amino acid from the CP43 subunit of the PS II enzyme forming coordination bonds with the Mn and Ca ions. Of these, D1-Asp170 and D1-Glu189 are key amino acid residues ligating the Ca. Therefore, changes in their geometry during the Kok cycle could indicate a change of the ligand environment of Ca and possibly the involvement of Ca in the shuttling of substrate waters into the cluster. In the S_1_ state XFEL structure (Kern *et al.*, 2018 ▸), Asp170 coordinates to Ca and Mn4 while Glu189 coordinates to Ca and Mn1 of the cluster. Note that in the structure by Umena *et al.* (2011 ▸), the Glu189-Ca distance is much longer (3.3 Å), suggesting no coordination. Figs. 4 ▸(*a*) and 4 ▸(*b*) show the sequence of changes of these two residues, in particular during the Kok cycle starting from the S_2_ state (no significant changes are observed going from S_1_ to S_2_). The omit map densities of the carboxyl­ate oxygens are also overlaid on the models. The first major change is observed in the 2F (50 µs) time point (Ibrahim *et al.*, 2020 ▸), where D1-Glu189 moves away from Ca (distance changes from 2.9 to 3.1 Å). As a consequence, this makes room for O_X_ to be inserted at the open coordination site of Mn1. Based on our observations, we previously hypothesized (Ibrahim *et al.*, 2020 ▸) that D1-Glu189, which is located close to the Tyr160–His191 residues, first senses the redox changes of this area, and this change then triggers the deprotonation and insertion of a water molecule (as OH) to Mn1 via the Ca coordination environment. Once detached from Ca in the S_2_ → S_3_ transition, Glu189 does not re-coordinate to Ca until very late in the subsequent S_3_ → S_0_ transition (Bhowmick *et al.*, 2023 ▸). This motion is seen in the 3F (2000 µs) time point where weak omit density of the oxygen close to Ca is observed. In all cases [except for a slight weakening in 2F (150 µs)] the coordination to Mn1 appears to be very rigid.

On the Asp170 side of the cluster, the most noticeable change during the S_2_ → S_3_ transition is a reduction of omit density of the Asp170 carboxyl­ate oxygen coordinating to Ca at 2F (150 µs) before being restored in the 2F (250 µs) time point. This time period matches that of the appearance of O_X_ density in the cluster (Fig. 3 ▸). A similar pattern can be seen in the S_3_ → S_0_ transition in the 3F (1200–2000 µs) time points during the recovery of density towards the S_0_ state. In this case, the time period seems to be in line with the release of O_2_ and subsequent cluster refilling with a new substrate water. Similar to the situation for Glu189, we do not see substantial changes in the omit density of the aspartate oxygen coordinated to Mn4, signifying a rigid Mn–carboxyl­ate interaction. A concomitant reduction of the density of the C_γ_ carbon of Asp170 is also observed in the aforementioned time points [example shown in Fig. 4 ▸(*c*)] which could indicate a twisting away of the carboxyl­ate arm coordinating the Ca while the oxygen ligated to Mn4 stays mostly in the same position. Hence our data suggest a possible temporary detachment of Asp170 from Ca during the water refilling (insertion) process and O—O bond formation phase of the Kok cycle [Fig. 4 ▸(*d*)]. The most probable entry point of the substrate water is via W4 and W3, both of which are coordinated to Ca (see discussion of water channels below). We postulate that the flexible coordination environment of Ca [it can ligate to 6–8 atoms, even 9 in rare cases (Katz *et al.*, 1996 ▸)] is suitable for such transient processes. It allows for controlled movement of water around the Ca ‘wheel’, reminiscent of proposals from early EPR studies (Tso *et al.*, 1991 ▸). In that study, Ca was suggested to be involved as a ‘gatekeeper’, limiting access to the highly oxidizing Mn in the OEC based on the changes observed in Ca depleted versus Ca containing PS II samples. Once refilling is complete, Asp170 can latch back on to the Ca and complete the coordination. As discussed above, the Glu189 interaction with Ca also changes on insertion (S_2_ → S_3_) and disappearance of O_X_ (S_3_ → S_0_). The picture that then starts to emerge suggests a crucial role for the Ca in water shuttling in both the S_2_ → S_3_ and the S_3_ → S_0_ transitions with well synced coordination changes with the protein environment. Such flexible yet well tuned interactions between the OEC and the protein environment appear to be an important strategy in the PS II active site.

### 3.3. Water and proton channels in PS II

**Figure 5:**
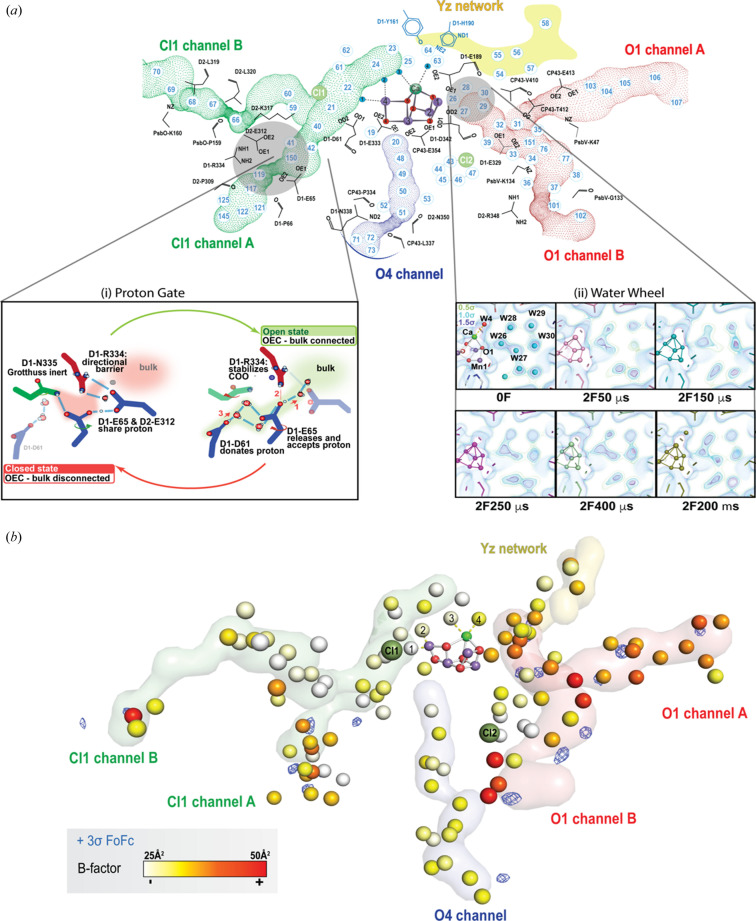
Water and proton channels in PS II. The Mn_4_CaO_5_ cluster in PS II is connected to the lumen of the thylakoid membrane by three distinct channels: the O1 channel, O4 channel and Cl1 channel. (*a*) Shown are the various waters and key amino acids that are found in these channels in the room-temperature crystal structures of PS II. Time-resolved XFEL data show the dynamics of the waters and the amino acids in these channels, correlated with changes in the Mn cluster. Two examples for the S_2_ → S_3_ transition are highlighted in the insets: (i) schematic of the proton gate in the Cl1 channel likely to be responsible for the transport of a proton to the bulk; and (ii) the electron density changes in the water wheel, adjacent to the Mn_4_CaO_5_ cluster, possibly a signature of water shuttling to the catalytic center. For the water wheel, the 2*mF*_o_ − *DF*_c_ map is shown at 3 contour levels (green: 0.5σ, light blue: 1σ, blue: 1.5σ) (*b*) The water molecules in a high-resolution room-temperature structure of PS II (1.89 Å, PDB entry 7rf1; Hussein *et al.*, 2021 ▸), colored by a gradient scale representing the *B* factor of each water (white for a value of 25 Å^2^ and red for a value of 50 Å^2^). The *mF*_o_ − *DF*_c_ map is overlaid in blue (showing only peaks >3σ). The O1 channel is colored in red, the O4 channel in blue and the Cl1 channel in green. Parts of the figure have been adapted from Ibrahim *et al.* (2020 ▸) and Hussein *et al.* (2021 ▸).

During the Kok cycle, two substrate waters need to be shuttled to the catalytic center and four protons need to be released to the lumen of the thylakoid membrane. Thus, water and proton pathways are an essential part of the PS II enzymatic machinery to facilitate efficient and well synchronized substrate delivery to and proton egress from the catalytic site. Several such channels have been proposed based on crystallography, spectroscopy as well as computational studies (Ishikita *et al.*, 2006 ▸; Murray & Barber, 2007 ▸; Ho & Styring, 2008 ▸; Gabdulkhakov *et al.*, 2009 ▸; Umena *et al.*, 2011 ▸; Vassiliev *et al.*, 2012 ▸; Frankel *et al.*, 2013 ▸; Weisz *et al.*, 2017 ▸; Kuroda *et al.*, 2021 ▸; Hussein *et al.*, 2021 ▸). The three main channels that are understood now to be crucial for water and proton transport in PS II are the O1 channel, Cl1 channel and the O4 channel. These channels are shown in Figs. 1 ▸(*a*) and 1 ▸(*b*). Note that these three channels (with species-specific variations) are observed in various oxygenic photosynthetic organisms despite being separated a long time ago in the evolutionary landscape (Hussein *et al.*, 2023 ▸). This signifies that the channels have a crucial role in the survival of these organisms, possibly related to the water-splitting reaction. Our room-temperature XFEL crystallography data have allowed us to follow the dynamics of these channels while undergoing catalysis. Fig. 5 ▸ shows the main structural changes we observed in the S_2_ → S_3_ transition. Based on our data, the O1 channel is likely to be the water intake pathway in this transition. This was partly based on the analysis of a high-resolution room-temperature structure of PS II (1.89 Å), obtained by averaging over the various S-states (Hussein *et al.*, 2021 ▸). We observed that the *B* factors were the highest in the O1 channel and also that it had several unmodelled *mF*_o_ − *DF*_c_ peaks throughout the channel, including near the catalytic center [Fig. 5 ▸(*b*)]. This is in contrast with the other channels that only exhibit similar *mF*_o_ − *DF*_c_ peaks near the bulk region. Additionally, the waters in the O1 channel also had the highest RMSD (root mean square deviation) in the various time points which points to a higher mobility of water in this channel. In fact, right next to the OEC in the O1 channel exists a water penta-cluster (W26–W30) that exhibits high variability in the electron density during various time points in the S_2_ → S_3_ transition [inset of Fig. 5 ▸(*a*)(ii)] (Ibrahim *et al.*, 2020 ▸; Hussein *et al.*, 2021 ▸). Such variability was interpreted as an indication that this penta-cluster could act as a ‘water wheel’ to deliver substrate water to the OEC.

For the release of the proton in the S_2_ → S_3_ transition, the Cl1 channel appears to be the most probable pathway from our structural data. In this channel, a rotation of D1-E65 (which is about 12 Å from the OEC) by about 50° is observed in our time-resolved data at 2F (150 µs) which leads to a complete re-arrangement of the hydrogen-bonded network. This residue then reverts back to its steady-state configuration in the next time point [2F (250 µs)]. Thus we hypothesize that there exists a proton gate region in the Cl1 channel [inset of Fig. 5 ▸(*a*)(i)] that controls the shuttling of the proton from the OEC to the bulk and minimizes back-reactions. The gate can exhibit two forms: a ‘closed’ state that does not allow proton egress and an ‘open’ state for efficient proton transfer to the bulk (Hussein *et al.*, 2021 ▸). The conformational changes at this gate region are closely intertwined with the electronic changes at the cluster with Mn oxidation and O_X_ insertion. This highlights the role of the broader protein environment in facilitating water-splitting and minimizing deleterious side reactions.

## 4. Conclusions

The advent of XFELs and their application to X-ray crystallography of biomolecules has created new opportunities to study enzymes under *operando* conditions, allowing us to bridge structure, dynamics and enzyme functionality. Much progress has been made since the first hard X-ray free-electron laser was introduced at the Linac Coherent Light Source (LCLS) in 2009 (Emma *et al.*, 2010 ▸). Since then, many XFEL facilities have become operational, *e.g.* SACLA (Japan), PALXFEL (South Korea), EUXFEL (Germany) and SwissFEL (Switzerland), underscoring the interest in using XFEL methods to study different types of scientific problems. In the past decade, simultaneous advances in XFEL science, sample delivery, sample preparation, detector technology and data processing have led to many remarkable findings in the biological sciences. These discoveries span a wide range of enzyme systems and timescales, highlighting the advantages of the femtosecond pulse of XFELs (*e.g.* Barends *et al.*, 2015 ▸; Kang *et al.*, 2015 ▸; Nogly *et al.*, 2018 ▸; Ishigami *et al.*, 2019 ▸; Srinivas *et al.*, 2020 ▸; Tetreau *et al.*, 2020 ▸; Dods *et al.*, 2021 ▸; Hosseinizadeh *et al.*, 2021 ▸; Nomura *et al.*, 2021 ▸; Rabe *et al.*, 2021 ▸). Our studies of PS II have driven several advances in the field and have uncovered key aspects of the inner workings of the catalytic machinery of the enzyme. By collecting time-resolved structural data, we can track the structural sequence of events in the enzyme through the catalytic cycle, for the mechanistic understanding of the water oxidation reaction. We can also track the structural dynamics of the protein surrounding the catalytic center, and how the orchestrated changes between the metal cluster and the protein and water environment are necessary in order to allow the reaction to proceed under ambient conditions. Some of the key findings from our XFEL studies of PS II are (1) the gradual insertion of a new water molecule (O_X_) in the Mn cluster in the S_2_ → S_3_ transition and the subsequent disappearance in the S_3_ → S_0_ transition when molecular oxygen is formed. (2) Well orchestrated structural dynamics of the ligating Asp170 and Glu189 residues that modulate coordination with the Ca of the OEC and possibly control water insertion. (3) The role of the water channels and the hydrogen-bonded network involved in substrate delivery and proton release. Such concerted modification of the protein environment, hydrogen-bond network and the OEC in PS II have allowed nature to drive the energetically uphill chemistry of water-splitting with minimum overpotential. Though we are starting to decipher some of the design rules used by nature to make these highly efficient catalysts, much work lies ahead to understand the interplay between the structural and electronic properties of the enzyme. By following these property changes along the reaction pathway, XFEL methods will help us construct a clearer picture of how enzymes such as PS II work and how we can rationally design synthetic systems to mimic these capabilities. This will be greatly beneficial for the development of sustainable energy technologies based on earth-abundant materials.
